# Clinical evaluation of deep learning–based clinical target volume three-channel auto-segmentation algorithm for adaptive radiotherapy in cervical cancer

**DOI:** 10.1186/s12880-022-00851-0

**Published:** 2022-07-09

**Authors:** Chen-ying Ma, Ju-ying Zhou, Xiao-ting Xu, Song-bing Qin, Miao-fei Han, Xiao-huan Cao, Yao-zong Gao, Lu Xu, Jing-jie Zhou, Wei Zhang, Le-cheng Jia

**Affiliations:** 1 Department of Radiation Oncology, 1st Affiliated Hospital of Soochow University, No. 188 Shizi Street, Suzhou, 215123 China; 2 Shanghai United Imaging Healthcare, Co. Ltd., Jiading, 201807 China; 3 United Imaging Research Institute of Innovative Medical Equipment, Shenzhen, 518045 China

**Keywords:** Cervical cancer CTV, Deep learning, Auto-segmentation, Registration

## Abstract

**Objectives:**

Accurate contouring of the clinical target volume (CTV) is a key element of radiotherapy in cervical cancer. We validated a novel deep learning (DL)-based auto-segmentation algorithm for CTVs in cervical cancer called the three-channel adaptive auto-segmentation network (TCAS).

**Methods:**

A total of 107 cases were collected and contoured by senior radiation oncologists (ROs). Each case consisted of the following: (1) contrast-enhanced CT scan for positioning, (2) the related CTV, (3) multiple plain CT scans during treatment and (4) the related CTV. After registration between (1) and (3) for the same patient, the aligned image and CTV were generated. Method 1 is rigid registration, method 2 is deformable registration, and the aligned CTV is seen as the result. Method 3 is rigid registration and TCAS, method 4 is deformable registration and TCAS, and the result is generated by a DL-based method.

**Results:**

From the 107 cases, 15 pairs were selected as the test set. The dice similarity coefficient (DSC) of method 1 was 0.8155 ± 0.0368; the DSC of method 2 was 0.8277 ± 0.0315; the DSCs of method 3 and 4 were 0.8914 ± 0.0294 and 0.8921 ± 0.0231, respectively. The mean surface distance and Hausdorff distance of methods 3 and 4 were markedly better than those of method 1 and 2.

**Conclusions:**

The TCAS achieved comparable accuracy to the manual delineation performed by senior ROs and was significantly better than direct registration.

## Highlights

- Deep learning has been used for the automatic contouring in cervical cancer.
- Adaptive radiotherapy responds to the morphologic changes and tumor anatomy.
- CT series acquired during treatment is required for creating new radiation plans.
- Automatically-obtained contouring has been comparable to manual delineation.
- The planning CT and corresponding contour can be used on the new CT series.

## Background

Cervical cancer is one of the most common cancers in worldwide, with incidence and mortality rates that rank fourth among all malignant tumors in females. Over 500,000 cases of cervical cancer are diagnosed each year, and most of these cases are in developing countries [1]. Because of the common use of cervical cancer screening in Western countries, the incidence of cervical cancer in these regions is decreasing slowly. However, in China, the incidence of cervical cancer continues to increase [2]. External beam radiation therapy, as well as brachytherapy, is an important component of cervical cancer therapy [3]. Radiotherapy can be used as a post-operative adjuvant treatment or as radical treatment, involving either internal or external irradiation.

Accurate contouring of the clinical target volumes is a key element of radiotherapy and is fundamental to maximizing the therapeutic ratio. However, the late toxicity rates associated with pelvic chemoradiation for cervical cancer are approximately 6%–23%. Therefore, reducing toxicity is critical, since some patients are young and toxicity may lead to many years of potentially debilitating conditions, such as incontinence, fistulae and malabsorption [4, 5].

The CTVs of cervical cancer are typically manually contoured and confirmed by ROs based on gynecological examination and surgery reports, as well as CT, magnetic resonance imaging (MRI) and other imaging. The definition of the target area depends on the clinician’s understanding and experience [6–8]. The quality, efficiency and repeatability of manual contouring vary among different ROs, and the time spent on contouring a patient is affected by the proficiency of ROs. In our clinic, target definition typically takes between 20 and 60 min. Automatic segmentation has been demonstrated to be an effective method to improve the consistency of contouring and to reduce operator effort [9, 10]. Currently, atlas-based automatic segmentation algorithms are widely used in commercial treatment-planning software. However, for organs and tumors that lack clearly defined boundaries or those with complex shapes, the results of segmentation are often unsatisfactory [11–13].

The DL-based method especially using convolutional neural network (CNN) has been proven as a promising technology for medical image segmentation. These DL-based segmentation algorithms have demonstrated significant advantages over classical medical image segmentation methods [14, 15]. Several groups have used DL to segment tumor targets that are not amenable to accurate contouring by traditional automatic methods. For example, Lin et al. contoured the gross tumor volume (GTV) of primary nasopharyngeal carcinomas based on MR images using a three-dimensional CNN. The authors reported an agreement between the algorithm segmentation result and the manually segmented reference dataset, in terms of DSC, of 0.79. In comparison, the between-manual-operator DSC showed a lower agreement, of 0.74. [16]. Men et al. applied a deep CNN to CT datasets of nasopharyngeal carcinoma cases for the segmentation of the primary tumor GTV, metastatic lymph node GTV, and the CTV. The DSC values were 0.809, 0.623 and 0.826, respectively, which compare favorably with both manual evaluations and the previously applied automatic methods [17]. Trebeschi et al. applied the DL method to the segmentation of rectal cancer from multiparametric MR images, and the DSC was 0.69 [18].

The purpose of this work was to determine the performance of the DL-based method in terms of accuracy, consistency and workflow acceleration for the auto-contouring and assisted manual contouring for adaptive radiotherapy in cervical cancer.

## Methods

### Dataset

Datasets were collected from 66 cervical cancer patients who received local radiotherapy at the Radiotherapy Department of the ****** from January 2017 to June 2019, 22 patients received radical radiotherapy and 44 patients received post-operative adjuvant radiotherapy. Each patient had contrast-enhanced CT scans for positioning and planning, and multiple plain CT scans were acquired during the treatment.

The datasets for each patient consisted of the following: (1) contrast-enhanced CT scan for positioning and (2) the related CTV contour, as well as (3) multiple plain CT scans during treatment and (4) the related CTV contour. After registration between the contrast-enhanced CT and plain CT scan for the same patient, a total of 107 cases were collected. This group included 30 radical radiotherapy cases and 77 post-operative adjuvant radiotherapy cases. In the 107 pairs of plain CT and contrast-enhancement CT scans, 92 pairs were randomly selected for the training set and the remaining 15 pairs were used as the test set. Among the 15 test set cases, 7 cases were radical radiotherapy and 8 cases were postoperative adjuvant radiotherapy.

**Fig. 1:**
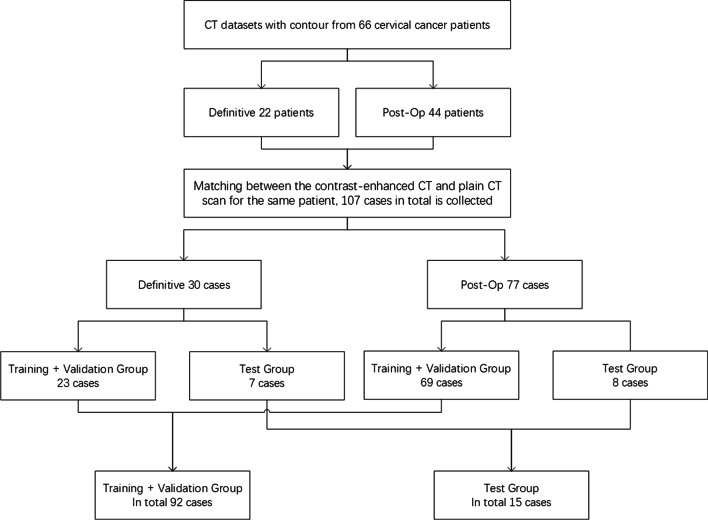
Details of the CT datasets

The distributions of patients and groups are shown in Fig. 1.

The CT scans covered the drainage area of pelvic lymph nodes, from L3 spine to the middle of the femur. The slice thickness of all scans is 3 mm. Based on the planning CT and during-treatment CT, the ROs contoured the clinical target area of conformed to the Radiation Therapy Oncology group (RTOG) [19] cervical cancer post-operative adjuvant radiotherapy target contouring proposal, the Japan Clinical Oncology Group (JCOG) [20] cervical cancer definitive radiotherapy external radiation target contouring standard and the International Federation of Gynecology and Obstetrics (FIGO) 2018 guide [21]. Definitive external pelvic irradiation included contour of the pelvic lymph drainage area dCTV1, as well as the parametrial area dCTV2, the GTV (including the primary focus and the pelvic positive lymph nodes), the cervix, the uterus, the upper third of the vagina, and the parametrial and pelvic lymph drainage area (not including the drainage area of inguinal lymph nodes and paraaortic lymph nodes). For post-operative auxiliary pelvic external irradiation, the pelvic lymph drainage area pCTV1 was delineated, including the pelvic lymph drainage area, the vaginal wall, and the upper third of the vagina. The uterosacral ligament, presacral lymph nodes and other potentially involved lymph nodes, and sufficient vaginal tissues (at least 3 cm below the margin) were also included. If no enlarged lymph nodes were detected in images, the external iliac lymph nodes, the internal iliac lymph nodes, the obturator lymph nodes and the presacral lymph nodes were included. In cases with a high risk of lymph node metastasis (such as in cases with a large tumor volume as well as suspected or determined low true pelvis internal lymph node metastasis), the total iliac lymph node area was also included; if total iliac or paraaortic lymph node metastasis was detected, the clinical target was extended, including the paraaortic lymph nodes. The upper boundary reaches the level of renal vessels and may need to extend further in the cranial direction to include the involved lymph nodes. For patients with infiltration of the lower third of the vagina, the bilateral inguinal lymph nodes were also covered.

**Fig. 2:**
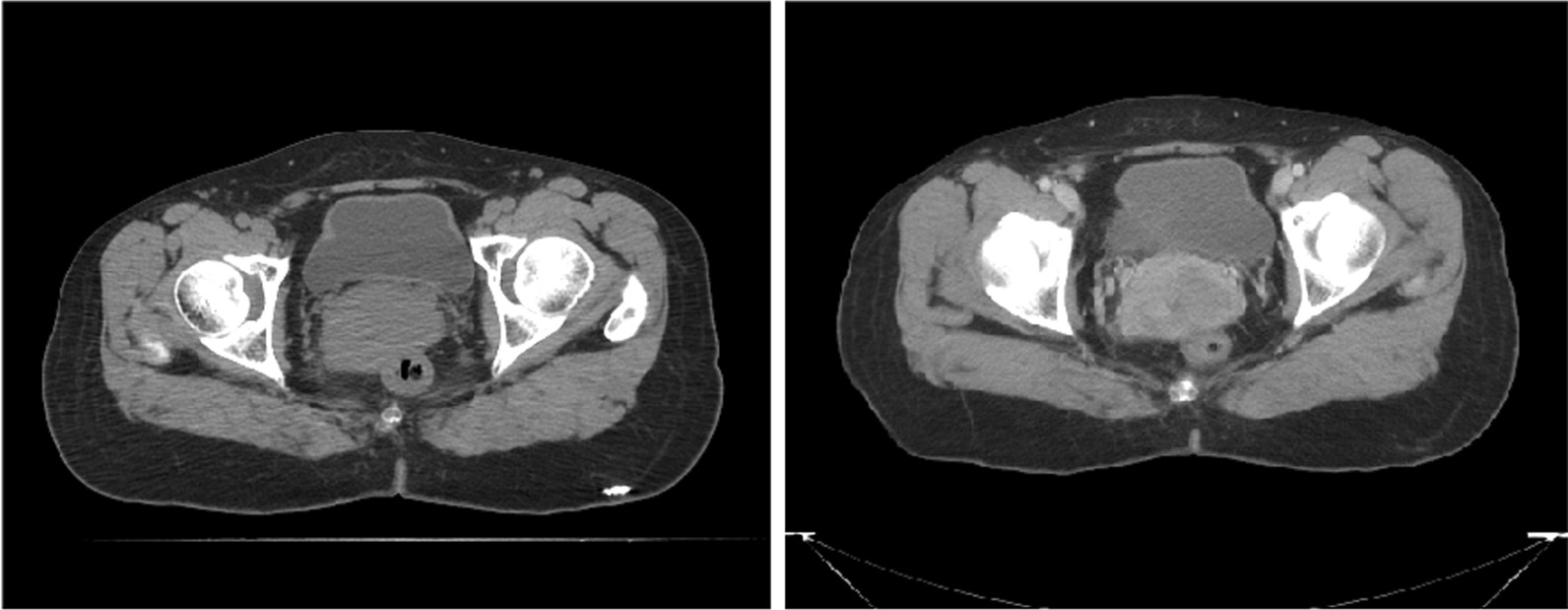
Difference of plain CT (left) and contrast-enhanced CT (right)

We used the CT series obtained during treatment to simulate the adaptive radiotherapy process retrospectively; the ROs contoured the clinical target volume on the during-treatment CT as well as on the planning CT. The contouring results were reviewed by the experts and then entered into this study. For simplicity, we did not distinguish between definitive cases and post-op cases, and the dCTV1 and pCTV1 were both treated as CTV1. The three input channels are the plain CT, contrast-enhanced CT and corresponding CTV contours. As shown in Fig. 2, the contrast-enhanced CT provides more details of organs, which can improve the performance of the segmentation network. The input CTV contours are 0 and 1 masks, which can provide initial weights of the input CT scans and help the segmentation network to locate the CTV.

### Four methods for CTV contouring on the during-treatment CT series

To create CTV contour on the during-treatment CT, the simplest approach is by directly copying the CTV contours from the planning CT to the during-treatment CT, either by a rigid registration (RR) or deformable registration (DR).

**Fig. 3:**
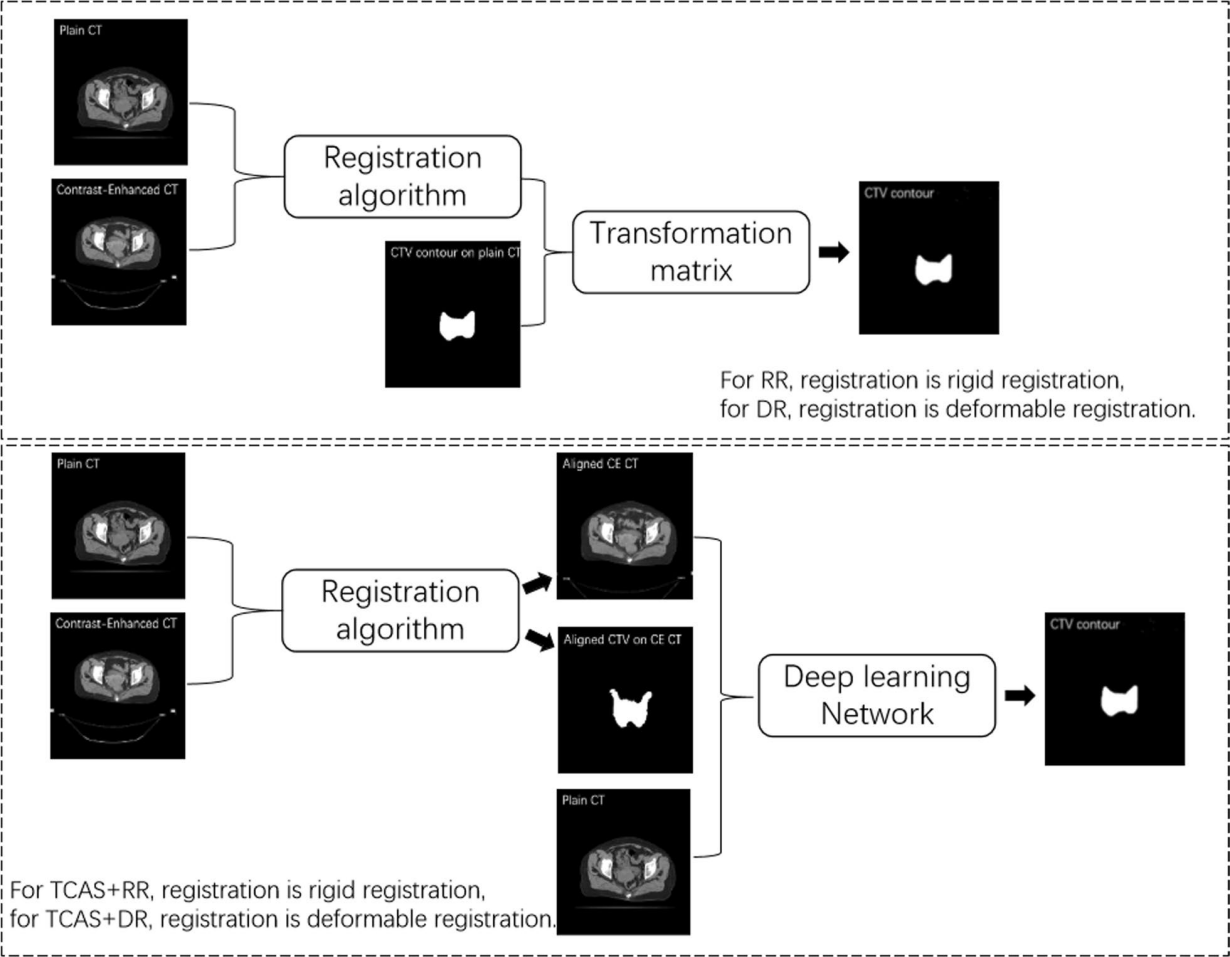
Workflows of four methods

In this manuscript. we proposed a TCAS method that uses information of the planning CT and the corresponding CTV contour. In this method, registration between the planning CT and the during-treatment CT is applied to align the CT series and the corresponding contour. The aligned planning CT (i.e. the contrast-enhanced CT) and the corresponding CTV contour are then used as another two channels of input to the DL network. Finally, the output of the network is the estimated CTV contour on the during-treatment CT (i.e. plain CT). The method that uses RR to align the planning CT and corresponding CTV contour is called TCAS + RR, while the method that uses DR for this alignment step is called TCAS + DR. The workflows of the four methods are shown in Fig. 3.

The only difference between the TCAS + RR and TCAS + DR is the registration algorithm component. For TCAS + RR, the algorithm first optimizes a 4 × 4 transformation matrix, which includes the 3 × 1 translation part and the 3 × 3 rotation part from the two images. This matrix is then applied on the image and the contour of the contrast-enhanced CT to create the aligned contrast-enhanced CT and aligned CTV on the contrast-enhanced CT. For TCAS + DR, the registration algorithm generates deformation vector fields, which have three components: fx, fy and fz. The field is then similarly applied on the image and contour of the contrast-enhanced CT.

### DL automatic segmentation network

DL-based methods require an initial training stage during which the neural network is provided with a large number of labeled 3D images. The CTV1 model was trained and validated using the 92 datasets in the training and validation group.

A three-dimensional VB-Net was used in TCAS + RR and TCAS + DR, but each implements a different spatial sampling regime. While the traditional V-Net algorithm [22] has achieved good results in many automatic segmentation studies, this algorithm often requires training a large model with a large number of parameters. A V-Net model file is generally about 250 MB, which not only leads to parameter redundancy, a waste of storage space and a reduction of calculation efficiency, but it also hinders the promotion and usage of automatic segmentation.

**Fig. 4:**
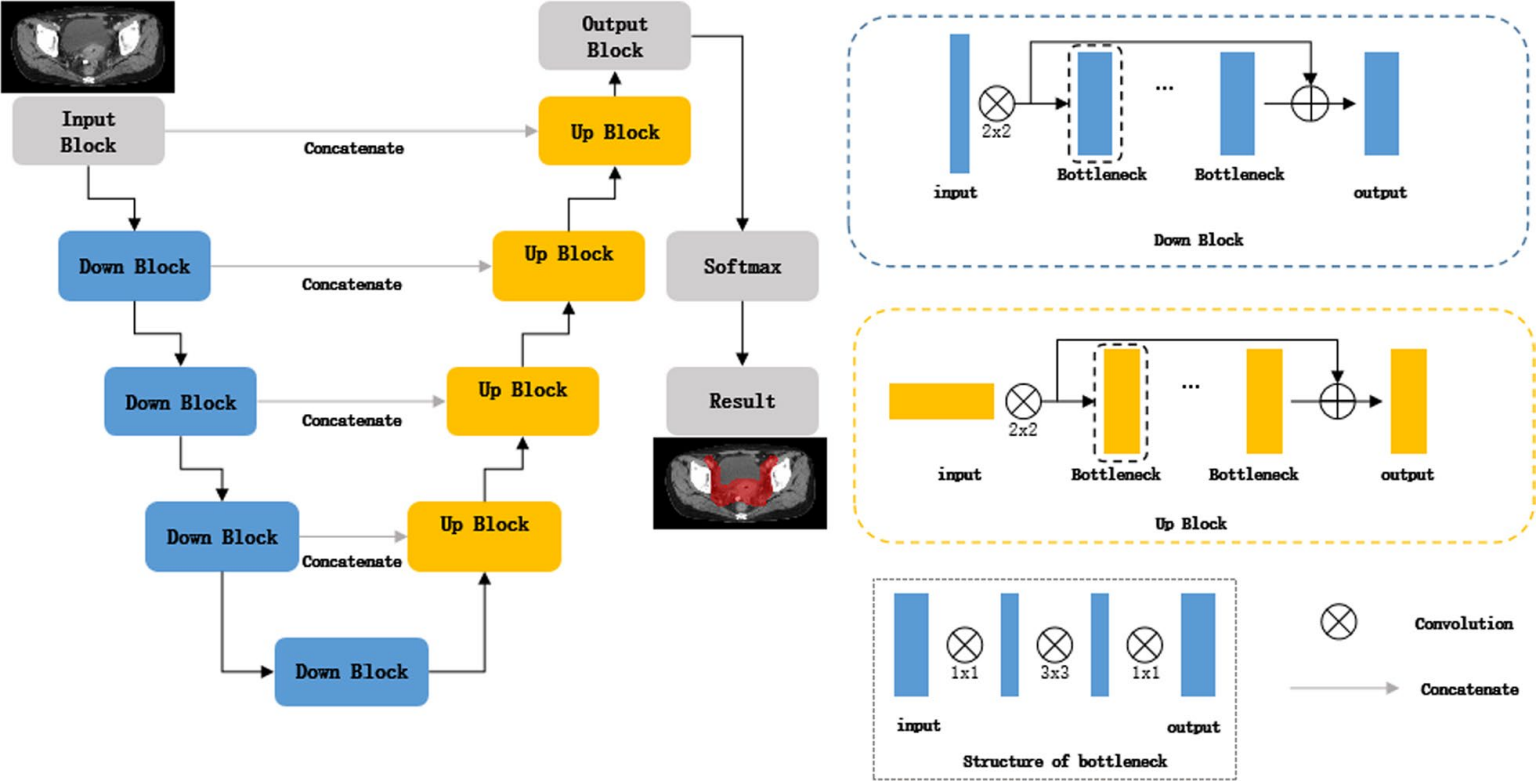
The structure of VB-Net

VB-Net, a new type of network structure, is proposed as an improvement over V-Net. The structure of VB-Net is shown in Fig. 4. The residual module in V-Net was designed using the concept of model compression. The convolution, normalization and activation layers in V-Net were replaced by a bottleneck structure in VB-Net. A bottleneck in a neural network is a layer with fewer neurons than its adjacent layers; this bottleneck encourages the network to compress feature representations to best fit in the available vector space. The bottleneck structure consists of three convolutional layers; the first and third convolutional layers, which use the unit convolution kernel, match the second (bottleneck) convolutional layer with the respective dimensions of the preceding and succeeding layers. The second convolution layer performs spatial convolution on the feature image that is reduced in dimension by the first convolution layer. Since spatial convolution is performed on the reduced dimension feature image, the number of model parameters may be significantly reduced, and this may lead to increased efficiency.

In pre-processing, global normalization was used for the plain CT and contrast-enhanced CT. We chose window level 40 and window width 700. The minimum and maximum CT values were − 310 and 390, respectively. CT values between these values were linearly normalized into the range [− 1, 1]. CT values less than the minimum were set to -1 and those greater than the maximum were set to + 1. For coarse model training, the images were resampled to [5 mm, 5 mm, 5 mm]. During fine model training, the images were resampled to [1 mm, 1 mm, 1 mm]. No data augmentation was applied. During post-processing, the maximum connected domain was extracted for CTV1. The learning rate is 1e-4, batch size is 6, patch size is [96, 96, 96] and the optimizer is Adam. The training hardware was Intel Xeon E5-2683 v3 with 64 GB memory and 4 NVIDIA Titan Xp. We trained 1000 epochs for 13 h. The predicting time was less than 2 s for one case. DSC was used for validation performance. The DSCs of TCAS + RR and TCAS + DR were 0.89 ± 0.02 and 0.90 ± 0.02, respectively.

### Registration methods

Image registration was performed for the two CT images acquired from the planning stage and the treatment stage. Generally, rigid and non-rigid registrations were sequentially performed and we used our in-house registration package. The in-house rigid registration is an optimization-based registration method. By using gradient decent optimization, the rigid transformation (translation and rotation) is estimated by minimizing the image dissimilarity. Since it is a mono-modal registration problem, the image dissimilarity metric is defined by sum of squared distance, and tri-linear interpolation is applied during optimization. To improve the efficiency, all the optimization procedures are implemented by using CUDA so that the registration efficiency can be significantly improved. For RR, a longitudinal semantic-aware registration algorithm was leveraged by two steps: (1) preprocessing the CT images of different time points and enhancing the salient anatomy information (e.g., bone area), and (2) performing longitudinal registration by image and semantic information using gradient decent optimization algorithms. A rigid transformation matrix was then obtained by the estimated translation and rotation parameters in 3D image space, and the CT image pair is globally aligned. After rigid registration, non-rigid registration was performed to further estimate the local deformations. An unsupervised DL framework was applied to directly estimate the deformation field from the two images after rigid registration [23]. In the training stage, the registration network is trained using the loss function defined by image dissimilarity (e.g., mean square error and normalized cross correlation) and regularization [24]. To further improve the smoothness and registration consistency, a new training strategy was used by introducing both pair-wise and group-wise deformation consistency constraints [25], in addition to the conventional similarity and topology constraints. Specifically, losses enforcing both inverse-consistency for image pairs and cycle-consistency for image groups were applied for training. After the model training, in the application stage, we directly obtain the deformation field by inputting the to-be-aligned image pair into the trained model; the registered CT image can be obtained by applying the rigid and non-rigid transformations.

### Qualitative and quantitative evaluation of algorithm accuracy

The CTV contour of the test group was created using the four methods described above: RR, DR, TCAS + RR and TCAS + DR. The algorithm accuracy was evaluated both qualitatively and quantitatively. For the RR and DR methods, RR and DR were applied to the test group, and CTV contours were generated according to the registration results. For the other two methods (TCAS + RR and TCAS + DR), the trained DL-based automatic segmentation CTV1 model was applied to the test group. We evaluated the segmentation using the dice similarity coefficient (DSC) [26], mean surface distance (MSD) [27] and the Hausdorff distance (HD) [28]. A better algorithm result will provide higher DSC and lower MSD/HD.

## Results

### Qualitative evaluation of algorithm accuracy

**Fig. 5:**
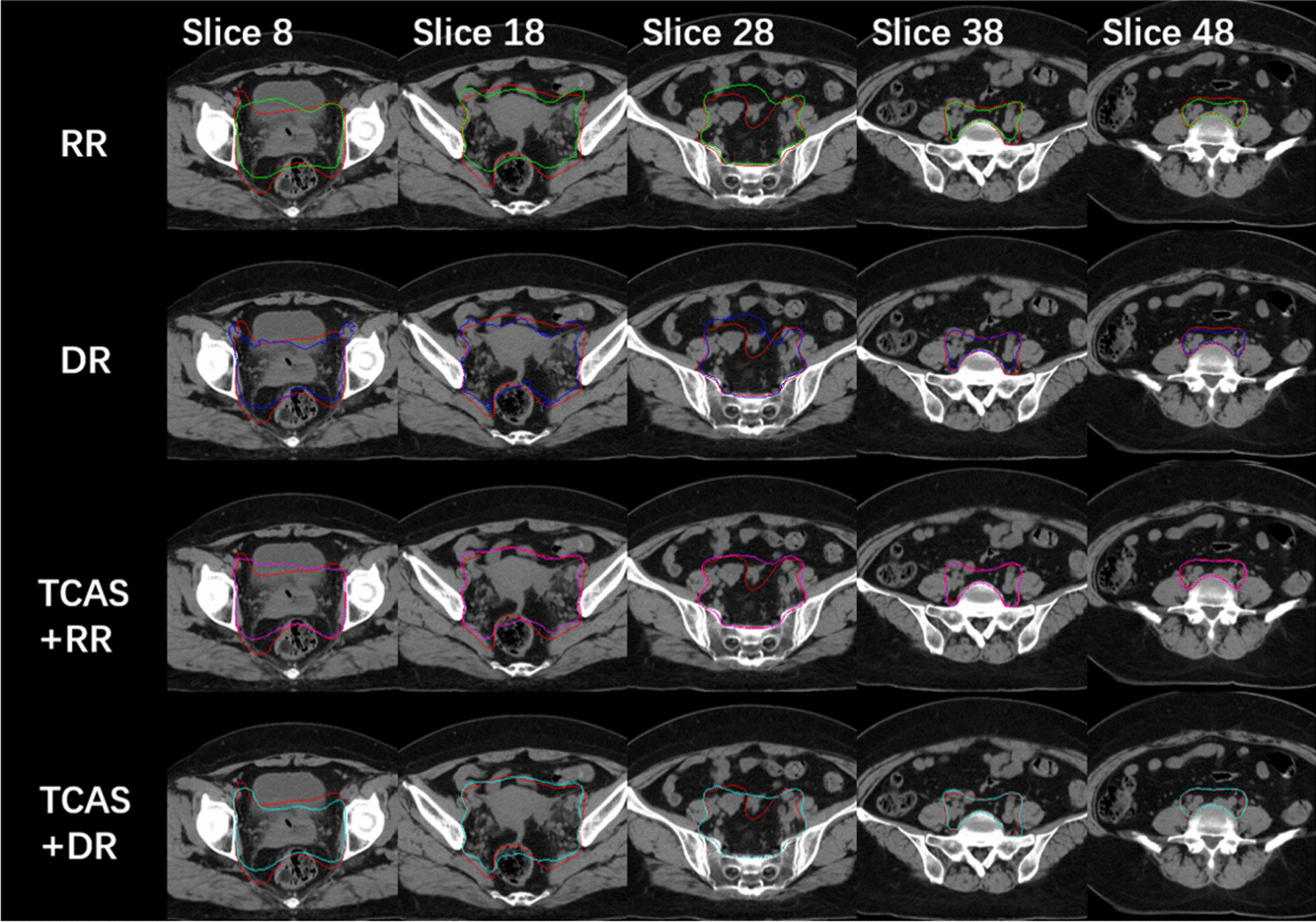
Contour of a representative test case using the five methods. Each column represents a different slice. The ground truth is in red, and the contour using the different methods is in the indicated colors. RR: Green, DR: blue, TCAS + RR: magenta, TCAS + DR: cyan

To evaluate algorithm accuracy, we randomly selected one case from the test group for analyses and performed contouring using each of the four methods. The contour results from the different four methods are shown in Fig. 5. Five typical slices with CTV contour were chosen to qualitatively evaluate the algorithm accuracy. The contour using TCAS + RR and TCAS + DR is significantly better than that of RR or DR.

### Quantitative evaluation of algorithm accuracy

We next used each of the four methods to generate CTV on plain CT findings in the test group, and the pCTV1 DL model was applied to the postoperative test group. The DSC, MSD and HD values were calculated for each model and are shown in Table 1. T test was used to compare between the groups. P values of DSC, HD and MSD were calculated separately between TCAS + DR and other methods. The performance of DSC between TCAS + DR and RR/DR was significantly different (p < 0.05). However, the sample size of the test group was the limitation of the statistical tests.

**Table 1:** Quantitative evaluation of algorithm accuracy (in the test group)

Method	DSC	P value	MSD (mm)	P value	HD (mm)	P value
RR	0.82 ± 0.04	2.04e−29	2.20 ± 0.75	0.018	8.23 ± 2.39	0.037
DR	0.83 ± 0.03	1.14e−6	2.03 ± 0.73	0.057	8.01 ± 2.44	0.065
TCAS + RR	0.89 ± 0.03	0.944	1.46 ± 0.84	0.997	6.14 ± 1.89	0.862
TCAS + DR	0.89 ± 0.02		1.45 ± 0.83		6.28 ± 2.31	

## Discussion

The typical CTVs for cervical cancer are usually large. The CTV position and shape are greatly influenced by the fill state of bladder, the rectum and other adjacent organs, which challenges the training of DL-based automatic segmentation models [27].

This study verified the clinical applicability of the DL-based automatic segmentation algorithm to evaluate the target area of cervical cancer in ART. The adaptive auto-segmentation algorithm achieved comparable accuracy to the manual delineation performed by senior ROs and was significantly better than direct registration.

In view of the high HD values observed in this study, we selected some of the results to analyze the differences between the automatic segmentation output and the reference contours. The inconsistencies were generally located around small lymph nodes at the level of the femoral head. During manual contouring process, the diagnosis information is used to determine whether these lymph nodes should be included. Therefore, performance could potentially be improved by the following: a) increasing the diversity of the training data (with and without included lymph nodes) and b) improving the consistency of the training data (for example, all lymph nodes are included or none are included). For example, Meng et al. [29] decreased the HD value of automatic segmentation result by post-processing. The HD value of automatic liver segmentation decreased from 89.2 to 29.2 mm, and the HD value of automatic liver cancer segmentation decreased from 65.4 to 7.7 mm. The direct HD value is generally used to represent the maximum difference between two contours and is very sensitive to abnormal contouring [30]. For automatic segmentation of the clinical target area, follow-up manual contouring and confirmation are generally needed, and these abnormal points can be easily modified. The use of MSD or 95% HD value may be better for the clinical evaluation of automatic segmentation results. In previous studies [23, 29] 95% HD was used to evaluate the accuracy of automatic segmentation, and the results are in the order of several millimeters. We calculated 95% HD values in the test group: 6.2 ± 2.6 mm, 13.7 ± 10.5 mm and 5.4 ± 1.9 mm for dCTV1, dCTV2 and pCTV1, respectively. For dCTV2, the 95% HD, like the HD, was relatively high. Therefore, the root cause of interoperator variation in contour defining needs to be addressed.

We also evaluated the consistency of the automatic segmentation and manual contouring results in the evaluation dataset. No significant difference was observed for dCTV1. However, the automatic segmentation results for dCTV2 and pCTV1, which were similar to the manual contours of senior ROs, were better than manual contours of the junior RO and one of the intermediate level ROs. In the current clinical workflow, manual contouring is typically first performed by junior and intermediate ROs, after which the contours are reviewed and modified by senior ROs. Improving the target contouring skills of junior personnel is critical. Importantly, our results suggest that the DL model will be useful to assist junior and intermediate ROs to improve the consistency and accuracy of their contouring, therefore reducing the time required by senior ROs to modify target areas.

The time required for manual contouring of a single target area was as great as 48 min in one case. It depends on the complexity of the target area, the size of the target area and the experience of the ROs. In comparison, the DL-based automatic segmentation method requires only a fraction of a second. In this regard, the automatic segmentation algorithm has a clear and significant advantage over manual contouring. The use of DL-based automatic segmentation model as an assisting tool will significantly reduce the time required for contouring the target area. In the follow-up study, we will evaluate and compare the time required for contouring by junior, intermediate and senior ROs with the help of DL-based automatic with the time required for manual contouring. Our study further indicates that DL-based autocontouring appears to be particularly well suited for cervical cancer evaluation, since the large CTV spans many CT slices, which would each need to be manually contoured. The sample size of the training and test groups is a limitation of the current research; we will evaluate the significance of the proposed method in large public datasets [31–33] and more patient data in the future.

The subjective evaluation results of ROs in the evaluation group show that for most autosegmentations, slight modifications by ROs are required before clinical use. These modifications are mostly because the automatic segmentation algorithm is currently not capable of following known fixed rules relating to specific boundaries. We believe these limitations will be addressed by including identified normal tissues and boundaries in the training data, so that the neural network is able to learn more general anatomic spatial relationships. Alternatively, a hybrid algorithm that combines DL with logical target area contouring rules can be developed.

## Conclusions

This study verifies the feasibility of application of the DL-based automatic segmentation method for cervical cancer radiotherapy in clinical practice. The results from automatic segmentation were consistent with that of reference contouring. Through comparative analysis of automatic segmentation results and manual contouring performed by three different groups of ROs, we conclude that the automatic segmentation results are in some cases equivalent to those of manual contouring by senior ROs. In addition, the time required for the automatic method is significantly shorter than that for manual contouring. For dCTV2, because of the small range of the target area and poor consistency of manual contouring results in the training and validation groups, the automatic segmentation results were relatively poor compared with the reference contouring. Similarly, the difference between manual contouring by different ROs was also large for dCTV2.

Based on the evaluation of nine ROs, the automatic segmentation results of most cases can be clinically applied, with only a few modifications required before application. In clinical application, implementation of the algorithm has reduced the time required for contouring and improved our clinical efficiency.

## Data Availability

The datasets generated and/or analysed during the current study are not publicly available due [none of the data types requiring uploading to a public repository are contained in this manuscript], but are available from the corresponding author on reasonable request.

## Abbreviations

DL: Deep learning
CTV: Clinical target volume
CTVs: Clinical target volumes
CT: Computed tomography
dCTV1: Delineate CTVs of the pelvic lymph drainage area
dCTV2: Delineate CTVs of parametrial area
pCTV1: The CTV of the pelvic lymph drainage area
DSC: Dice similarity coefficient
MSD: Mean surface distance
HD: Hausdorff distance
AI: Artificial intelligence
BT: Brachytherapy
EBRT: External beam radiation therapy
IMRT: Intensity-modulated radiation therapy
ROs: Radiation oncologists
MRI: Magnetic resonance imaging
CNNs: Convolutional neural networks
GTV: Gross tumor volume
GTV-nx: The primary tumor GTV
GTV-nd: Metastatic lymph node GTV
RTOG: Radiation Therapy Oncology Group
JCOG: Japan Clinical Oncology Group
